# Clinical relevance of double heterozygosity revealed by next-generation sequencing of homologous recombination repair pathway genes in South African breast cancer patients

**DOI:** 10.1007/s10549-024-07362-2

**Published:** 2024-05-30

**Authors:** Nerina C. van der Merwe, Ines Buccimazza, Bianca Rossouw, Monica Araujo, Kholiwe S. Ntaita, Mardelle Schoeman, Karin Vorster, Kgabo Napo, Maritha J. Kotze, Jaco Oosthuizen

**Affiliations:** 1 Division of Human Genetics, Faculty of Health Sciences, University of the Free State, Bloemfontein, South Africa; 2 Division of Human Genetics, National Health Laboratory Service, Universitas Hospital, Bloemfontein, South Africa; 3 Genetics Unit, Inkosi Albert Luthuli General Hospital, Durban, South Africa; 4 Department of Surgery, Nelson R Mandela School of Medicine, Inkosi Albert Luthuli General Hospital, Durban, South Africa; 5 Division of Human Genetics, National Health Laboratory Service, Braamfontein, Johannesburg, South Africa; 6 Division of Human Genetics, Faculty of Health Sciences, University of the Witwatersrand, Johannesburg, South Africa; 7 Division of Molecular Biology and Human Genetics, Faculty of Medicine and Health Sciences, Stellenbosch University, Cape Town, South Africa; 8 Department of Oncology, Free State Department of Health, Universitas Annex Hospital, Bloemfontein, South Africa; 9 Department of Oncology, Faculty of Health Science, University of the Free State, Bloemfontein, South Africa; 10 Division of Chemical Pathology, Department of Pathology, National Health Laboratory Service, Tygerberg Hospital, Cape Town, South Africa; 11 Division of Chemical Pathology, Department of Pathology, Faculty of Medicine and Health Sciences, Stellenbosch University, Cape Town, South Africa

**Keywords:** Breast cancer, Double heterozygotes, Homologous recombination repair, Clinical relevance

## Abstract

**Purpose:**

Genetically predisposed breast cancer (BC) patients represent a minor but clinically meaningful subgroup of the disease, with 25% of all cases associated with actionable variants in *BRCA1/2*. Diagnostic implementation of next-generation sequencing (NGS) resulted in the rare identification of BC patients with double heterozygosity for deleterious variants in genes partaking in homologous recombination repair of DNA. As clinical heterogeneity poses challenges for genetic counseling, this study focused on the occurrence and clinical relevance of double heterozygous BC in South Africa.

**Methods:**

DNA samples were diagnostically screened using the NGS-based Oncomine™ BRCA Expanded Research Assay. Data was generated on the Ion GeneStudio S5 system and analyzed using the Torrent Suite™ and reporter software. The clinical significance of the variants detected was determined using international variant classification guidelines and treatment implications.

**Results:**

Six of 1600 BC patients (0.375%) tested were identified as being bi-allelic for two germline likely pathogenic or pathogenic variants. Most of the variants were present in *BRCA1/2*, including two founder-related small deletions in three cases, with family-specific variants detected in *ATM, BARD1, FANCD2, NBN,* and *TP53*. The scientific interpretation and clinical relevance were based on the clinical and tumor characteristics of each case.

**Conclusion:**

This study increased current knowledge of the risk implications associated with the co-occurrence of more than one pathogenic variant in the BC susceptibility genes, confirmed to be a rare condition in South Africa. Further molecular pathology-based studies are warranted to determine whether clinical decision-making is affected by the detection of a second pathogenic variant in *BRCA1/2* and *TP53* carriers.

## Introduction

Patients with a genetic predisposition to breast cancer (BC) represent a minor but clinically meaningful subgroup of the disease. This predisposition accounts for 5–15% of the detected malignancies. It is mainly due to germline likely pathogenic (LP) and pathogenic variants (PVs) in high- and moderate-penetrance genes such as *BRCA1* and *BRCA2*, which are involved in homologous recombination repair (HRR) of DNA damage [1]. The frequency of causative variants in these two genes varies and is influenced by specific geographical locations, unique population lineages, and some non-genetic factors. In populations such as these, founder effects may exist, resulting in a higher incidence of causative variants. This could result in more patients being bi-allelic or double heterozygous (DH) for LP/PVs. This is the case for the Ashkenazi Jewish population, where the DH percentage is 1.8% compared to 0.2–0.8% in different non-founder ethnic groups [2]. Based on these low percentages, the probability of carrying more than one PV in a single patient is generally low. Although rare, their presence could result in notable clinical variability in patients depending on the genes involved and their position or role in the HRR pathway.

Le Page et al. [2] reported on various studies that described DHs in the *BRCA* genes observed in hereditary breast and ovarian cancer syndrome (HBOC) families. With the advent of next-generation sequencing (NGS), more patients are currently being screened for various genes associated with HBOC. More recent reports indicated the involvement of various non-*BRCA* genes in critical pathways such as DNA repair [3, 4]. Little, however, is known about their phenotype. As clinical heterogeneity among families poses great challenges for genetic counseling when dealing with hereditary cancer syndromes, this study focused on the occurrence and clinical relevance observed of DH BC patients identified in the South African (SA) population using NGS-based panel testing.

## Methods

DNA was extracted from peripheral blood, where after the samples were screened using the NGS-based Oncomine™ BRCA Expanded Research Assay (Life Technologies) according to the detailed protocol described by van der Merwe et al. [5]. Variants were filtered and annotated using the Ion Reporter™ Software version 5.18.2 (Thermo Fisher Scientific Inc., USA). The average coverage depth was 936X.

The clinical significance of the variants was determined based on the updated guidelines of the American Society of Medical Genetics and Genomics and the Association for Molecular Pathology [6], including the amended version designed specifically for *TP53* [7]. Freely accessible public databases such as ClinVar, ClinGen, LOVC, BRCA Exchange, and the genomic search engine VarSome [8] were used to interrogate and ultimately classify variants.

### Patients

Samples of 1600 patients were received at the National Health Laboratory Service (NHLS) Human Genetics laboratory in Bloemfontein, SA. The patients were diagnosed with breast, ovarian or prostate cancer, for whom clinicians requested diagnostic multigene panel screening to confirm/exclude HBOC. All the patients underwent pre-and post-test counseling, during which clinical and family-related cancer information was shared after obtaining informed consent for diagnostic testing, data sharing, and publication of results. Patients' unique identifiers were de-identified for publication purposes.

## Results

Six female patients (0.375%) were identified as being bi-allelic for two germline LP/PVs. The genetic variants, together with patient and tumor characteristics, are summarized in Table 1. The average age at diagnosis was 37.5 years. Although all the patients reported a family history of breast and/or various cancer types, *Cases 5&6* had the highest risk based on the presence of multiple first-degree relatives affected by the disease. A high percentage of *BRCA1/2* variants, as expected for HBOC syndrome, were observed (50%, 6/12). Of the non-*BRCA* genes, *TP53* was observed for two cases, with the remaining genes (*ATM*, *BARD1*, *FANCD2,* and *NBN*) all contributing a single variant (Table 1). The family history of *Case 1* exhibited the widest range of cancer types, as expected from a pathogenic *TP53* variant, with a family member diagnosed with a cancerous lesion on the chin and deceased at age 29.

**Table 1:** Clinical details for each case

Characteristic	Case 1	Case 2	Case 3	Case 4	Case 5	Case 6
Genetic result						
LP/PV 1	BARD1c.448C > T (p.Arg150Ter)	BRCA1 c.1374del (p.Asp458Glufs*17)	BRCA1 c.45dup (p.Asn16Ter)	TP53 c.523C > T (p.Arg175Cys)	BRCA1 c.1374del (p.Asp458Glufs*17)	ATM c.7978G > T (p.Glu2660Ter)
LP/PV 2	TP53c.695_696insT (p.His233Profs*7)	BRCA2c.7934del (p.Asp2645Asnfs*3)	BRCA2c.7934del (p.Asp2645Asnfs*3)	FANCD2c.2983G > T (p.Gly995Ter)	NBN c.896 + 2 T > C Splice variant	BRCA2c.516G > A Splice variant
Breast cancer diagnosis						
Age at dx	24	33	47	46	36, 48	39
Clinical symptoms prior to dx	Tender mobile lump	Not indicated	Palpable lump	Palpable lump	Palpable lump	Palpable lump
Method used for dx	Fine needle aspirate	Not indicated	Core biopsy	Core biopsy	Core biopsy	Core biopsy
Laterality	Right breast	Right breast	Left breast	Right breast	Bilateral, metachronous	Right breast
Characteristics of tumor						
BIRADS classification	BIRADS V	Not indicated	BIRADS IV	BIRADS V	Not available	BIRADS V
Tumor grade (Modified Bloom and Richardson)	Grade III	Not indicated	Grade III	Grade II	Grade III	Grade III
Tumor classification	cT2 cN0 M0	cT3 cN0 M0	cT3 cN2 M0	cT4b cN1 M0	Not available	cT4b cN1 M0
Stage	Stage IIa	Stage IIb	Stage IIIa	Stage IIIb	Not available	Stage IIIb
Cancer type	IBC NST	IBC NST	IBC NST	IBC NST	IBC NST	IBC NST
DCIS present	Yes, high grade	No	No	Yes	Not available	Yes
Number of lymph nodes involved	0/4	0/6	0/10	1/6	Not available	0/5
Lymphovascular invasion	Absent	Absent	Absent	Absent	Absent	Absent
Family history						
Yes/No	Yes	Yes	Yes	Yes	Yes	Yes
Family history description						
Breast cancer	n/a	M (dx30)	MGA (dx 40 s)	PGM (dx?) MA (dx60) MC (dx45)	S (dx40;41) S (dx55)	M (dx50) S (dx47)
Prostate ca	PU (dx > 60)	n/a	PGU (dx 60 s)	n/a	n/a	n/a
Pancreatic ca	n/a	n/a	MGM (dx 65)	n/a	n/a	n/a
Melanoma	n/a	n/a	MGA (dx?)	n/a	n/a	n/a
Other cancer types	Thyroid (dx58) Cervical: (dx40s) Mouth: (dx29)	n/a	Stomach (dx?)	M (dx?) Colon ca: MA (dx < 55)	n/a	n/a
Immunohistochemistry						
					Left breast; Right breast	
ER	Negative	Negative	Negative	Positive	Negative; Negative	Positive
PR	Negative	Negative	Negative	Positive	Negative; Negative	Positive
HER2	Negative	Negative	Negative	Positive	Negative; Negative	Negative
Ki67%	50%	Not available	40%	70%	70%; not available	Not available
Treatment						
Neoadjuvant therapy	Yes	Yes	Yes (defaulted)	Yes	No	Yes
Adjuvant therapy	Yes, five cycles	Yes	Yes	No	Yes	Yes
Therapeutic effect	Sataloff TA	Sataloff TA	Sataloff TA	Sataloff TA	No	Sataloff TB
Radiotherapy	Yes	Not indicated	Yes (left chest wall)	Yes	Yes	Yes (right chest wall + boost)
Hormone therapy	Yes	Not indicated	No	Yes	No	Yes (5–10 years)
Surgery received						
Oophorectomy	No	Yes	No	No	n/a	No
Radical mastectomy	No	Yes (right breast)	Yes (left breast)	Yes	n/a	Yes
Segmentectomy/lumpectomy	Yes	No	No	No	Yes	No
Contralateral mastectomy	No	Yes (prophylactic left)	Yes (prophylactic right)	No	n/a	No
Nipple conservation	Yes	No	No	No	n/a	No
Segregation studies						
Yes/No	Yes M: BARD1–/TP53–	No	Yes S: BRCA1 + /BRCA2– D: BRCA1–/BRCA2–	No	Left breast; Right breast Yes D: BRCA1 + /NBN not tested Son: BRCA1–/NBN not tested Son: BRCA1 + /NBN not tested	No

LP - likely pathogenic, PV - pathogenic variant, PU - paternal uncle, M - mother, PGM - paternal grandmother, MA - maternal aunt, MC - maternal cousin, S - sister, D - daughter, MM - multiple myeloma, ca - cancer, dx - diagnosis, IBCNST - invasive breast carcinoma of no special type, DCIS - ductal carcinoma in situ, ER - estrogen receptor, PR - progesterone receptor, HER2 - human epidermal growth factor receptor 2, *Ki-67%* - the % of tumor cells positive for Ki-67 nuclear staining, Sataloff TA - total or near total therapeutic effect, Sataloff TB - 50% therapeutic effect, but less than TA, n/a - not applicable

Most of the tumors were detected using mammography, with their BIRADS classification ranging from suspicious to highly suggestive of malignancy (Table 1). Five of the seven tumors (*Case 5* was diagnosed with bilateral synchronous BC) were triple-negative (TNBC), with the remaining two representing a potential luminal molecular subtype identified by immunohistochemistry and FISH for Her2 equivocal cases. Most of the tumors were of grade III, with two of the tumors already progressed to stage IV. All but one patient received neoadjuvant therapy, although *Case 3* defaulted after only three of the planned eight cycles due to adverse side effects. Despite the reduced cycles, the tumor responded adequately to perform surgery. All but one of the TNBC cases achieved a pathologic complete response (pCR) post neoadjuvant therapy, thereby improving their outcome and reducing the recurrence risk (Table 1).

For *Case 1*, patient autonomy was respected, as she opted for breast-conserving therapy due to her young age. A segmentectomy of the right and a reduction of the left breast were performed. Upon disclosure of the genetic test result, the patient was advised to consider a bilateral mastectomy with or without reconstructive surgery. However, the patient defaulted from returning to the clinic for treatment. Of the three *BRCA1* mutation carriers, only *Case 2* thus far has opted for a risk-reducing bilateral salpingo-oophorectomy (BSO) as part of her treatment plan. *Case 3* has recently completed adjuvant treatment and has been referred to discuss and plan her BSO. Segregation and predictive testing of these variants in related family members has only recently been initiated and is underway (Table 1).

## Discussion

A mere 0.375% of the patients were bi-allelic for LP/PVs in genes involved in the HRR pathway. Although multiple international reports described DHs for both *BRCA1* and *BRCA2* before [9–11], their combination with non-*BRCA* genes is limited [3, 4]. The likelihood of being DH for LP/PVs in different dominant cancer-predisposing BC genes is relatively uncommon but is increased in populations where founder effects exist [4], such as SA [12, 13].

Identifying germline LP/PVs in genes associated with hereditary BC has implications for the index patient and related family members, as preventative measures and management strategies can be implemented for mutation carriers to prevent or enhance early cancer detection. The presence of specific variants can impact and change a patient’s treatment plan, for example, the use of poly(ADP-Ribose) polymerase (PARP) inhibitors associated with increased cancer cell sensitivity [14].

## Molecular interpretation

### *BARD1* and *TP53 - Case 1*

*BARD1*(NM_000465.4):c.448C > T (p.Arg150Ter) is predicted to cause a loss of normal protein function through either protein truncation or nonsense-mediated mRNA decay. The variant is associated with a personal and/or family history of BC and OVC [15, 16]. Together with *BRCA1*, the two tumor suppressor proteins form a heterodimeric complex that mediates cell survival and repair of DNA damage by maintaining genomic stability [17]. This occurs through nuclear functions such as DNA damage signaling and repair, transcriptional regulation, and cell cycle control [18]. The ineffective *BARD1* protein will lack the critical ankyrin repeat and BRCT domains, thereby abolishing the protein’s interaction with p53 and *BARD1*-p53-dependent apoptosis [19].

TP53(NM_000546.6):c.695_696insT (p.His233Profs*7) occurred in the most frequently mutated region (exons 5–8), which forms part of the DNA-binding domain [20, 21]. When intact, the p53 protein functions as a cell stress-activated sequence-specific transcription factor that regulates gene expression in essential cellular processes [22]. The highly conserved DNA-binding domain, which includes exon 7, results in the formation of a stable protein with tumor suppressor activities that include the induction of cell cycle arrest, apoptosis, or DNA repair [22, 23]. With premature truncation, the protein will lack canonical p53 tumor suppressor activities, promoting cancer cell proliferation, survival, and potentially metastasis.

### *BRCA1* and *BRCA2 *- *Cases 2* and *3*

Two truncating variants, namely BRCA1(NM_007297.4):c.1374del (p.Asp458Glusfs*17) and BRCA2(NM_000059.4):c.7934del (p.Arg2645Asnfs*3) were observed for *Case 2*, with BRCA1 (NM_007297.4):c.45dup (p.Asn16Ter) and *BRCA2* c.7934del detected for *Case 3* (Table 1). Of these, *BRCA1* c.1374del and *BRCA2* c.7934del represent confirmed SA founder variants [12, 24, 25], with the third representing an SA recurrent variant (*BRCA1* c.45dup) [26]. These two cases currently represent the third and fourth occurrence of DH involving founder variants in *BRCA1/2* in SA [9].

Although *BRCA1/2*’s association with breast and OVC was confirmed two centuries ago [27, 28], their involvement in other cancer types, such as prostate and pancreatic cancer [29, 30], has recently come to the forefront. These two proteins play a crucial role in maintaining genomic integrity during DNA double-strand break (DSB) repair by regulating repair [31, 32] through interaction with numerous other proteins involved in the HRR machinery [33].

*BRCA1* is involved in the recognition of DNA breaks and is responsible for the recruitment of *BRCA2* to the break, where they interact with PALB2 [34]. As exons 11–13 encode the central regions of the protein, truncating variants in the early parts of the gene (*Case 3*-exon 2; *Case 2*-exon 10) will result in the loss of multiple binding domains, impacting *BRCA1*’s function and expression.

To ensure the maintenance of genomic stability upon replication stress, *BRCA2* regulates the stalled DNA replication fork by loading and stabilizing polymerized RAD51 onto the DNA. This is facilitated through binding to its BRC repeats, situated toward the C terminal of the protein [35]. In the case of *BRCA2* c.7934del, although the RAD51 binding domain constituting the BRC repeats (exon 11) will be intact, the secondary DNA-binding domain (exons 12–26) will be absent, resulting in DNA damage repair defects and tumorigenesis.

### *TP53* and *FANCD2 - Case 4*

TP(NM_000546.6):c.523C > T (p.Arg175Cys) in exon 5 is also situated in the DNA-binding domain. The Arg-175 residue is a mutational hotspot for hypomorphic variants and is involved in the structural integrity of the p53 DNA-binding domain. The Arg-175 residue forms part of the hydrophobic L3 hairpin that assists in stabilizing the DNA-binding domain, which is affected by the introduction of the new hydrophilic Cys-175 residue [36, 37].

*FANCD2* forms part of the Fanconi Anemia (FA) pathway, an uncommon heterogeneous biallelic genetic disorder characterized by progressive aplastic anemia, congenital growth malformations, and tumor susceptibility [38]. This pathway involves more than 22 identified genes and plays a critical role in the repair of genomic instability such as repairing DNA interstrand cross-links needed for homologous DNA repair. Failure to repair these cross-links are closely associated with tumorigenesis [39].

*FANCD2*, together with *BRCA2*, is essential for protecting stalled replication forks against nucleolytic degradation through the regulation of responses to replication stress induced by mitomycin and hydroxyurea [40, 41]. Apart from the two DNA-binding domains, the EDGE domain (aa 1427–1430) is in the carboxyl terminus encoded by exon 44. This domain is indispensable for cellular resistance to mitomycin, as it stimulates interstrand crosslinks [42], which prevent DNA unwinding by DNA helicases and stop the progression of the replication and transcription machinery [43, 44].

The truncating FANCD2(NM_033084.6):c.2983G > T (p.Gly995Ter) variant falls within the second DNA-binding domain. The PV variant will not only impact this domain but will also result in the abolition of the EDGE domain. As the interstrand crosslinks are difficult to repair, the absence of a full-length protein, including the indispensable EDGE domain, will increase replication stress and mitomycin’s upregulation. It will ultimately result in more interstrand crosslinks associated with no DNA repair and a predisposition to cancer due to the inactivation of the cell cycle barriers.

As multiple recognized BC genes such as *BRCA1* (also known as *FANCS*), *BRCA2* (*FANCD1*), and *PALB2* (*FANCN*) all form part of both the FA pathway and the HRR machinery needed for double-stranded DNA repair, various studies investigated the role of other FA genes such as *FANCD2* with regards to cancer susceptibility, and more specifically BC [45]. Despite numerous studies reporting sensitivity of FA mutation positive tumors being sensitive to DNA damaging agents such as PARPi due to the accumulation of DNA lesions, no consensus on this matter has been reached, mainly due to either small sample sizes or a lack of molecular characterization in previous epidemiological studies [45–47].

Recently, Zhu and co-workers [48] screened 1481 Chinese high-risk BC patients for 16 FA genes and reported 38 pathogenic mutation carriers in the 12 unconfirmed BC genes after excluding *BRCA1*, *BRCA2*, *PALB2,* and *RAD51C*. Of these genes, *FANCA* was the most commonly mutated (0.54%) followed by *FANCD2* (0.41%). Upon investigation, various clinicopathological associations (although not statistically significant, possibly hampered by the small sample size) were reported, pertaining to larger tumor sizes, lower ER and PR positivity rates and lower 3.5 year invasive disease-free survival and distant recurrence-free survival rates in mutation carriers when compared to non-carriers [48].

The risk to mono-allelic *FANCD2* carriers to develop cancer were investigated using the relatives of patients with FA enrolled in the National Cancer Institute Bone Marrow Failure Syndrome cohort [49]. Patients with cancer and their unaffected relatives were genotyped where after the rates, types of cancer and their age at diagnosis were evaluated. Although the study indicated that the risk of cancer was not increased among all FA heterozygotes, only two genes were conclusively excluded as potential risk genes, namely *FANCA* and *FANCC* (observed versus expected ratios (O/E) of 0.92 and 0.91, respectively). With regards to *FANCD2*, the O/E for cancer risk and more specifically BC risk, were similar to that of *BRCA2* (*FANCD1*), an established high-risk BC gene. The O/E for all cancer types were 2.8 for *BRCA2* and 2.85 for *FANCD2*, with the risk for BC specifically indicated as 11.0 and 16.3, respectively [49].

Studies evaluating the protein expression and ubiquatination levels of FANCD2 in breast tumors have also identified an association between BRCA1 (another confirmed high-risk BC gene) and FANCD2 protein expression, and in breast tumors an association between FANCD2 protein expression and tumors size, TNM stage, ER status and Ki-67 index and ultimately, disease-free survival rate. Multivariable analysis demonstrated that high levels of FANCD2 expression and low levels of FANCD2 ubiquitination can be considered as independent prognostic factors and of value to BC patients [50–52]. These studies suggest that pathogenic variants in *FANCD2* would abolish the protective effect of FANCD2 during tumorigenesis when higher expression levels is observed [50–52].

Although mono-allelic disruptive variants in *FANCD2* is rare, they are now more frequently identified during genetic testing, emphasizing the need for a thorough understanding of the function and epidemiology of this gene with regards to cancer and more specifically BC risk. For these reasons, the case was included in this report despite the fact that there are currently no consensus risks and management options for the disease for mono-allelic mutation carriers.

### *BRCA1* and *NBN *- *Case 5*

Apart from the *BRCA1* Afrikaner founder variant, an LP splice site variant was identified in NBN(NM_00248.5):c.896 + 2 T > C (p.?). The change affects a rare donor splice site, which is expected to disrupt RNA splicing and result in protein function loss. The nibrin protein forms part of and regulates the activity of the MRN protein complex involved in the recognition of and coordination of DSB repair through interaction with ATM. Nibrin guides this complex, carrying the two proteins to the DNA-damaged site in the cell’s nucleus [53], where the complex proceeds to mend the damage [54].

Disruptive variants in *NBN* lead to the autosomal recessive Nijemen breakage syndrome associated with several types of disease, particularly cancer [55]. As earlier studies suggested an association with BC (relative risk of 2.7) [56], it resulted in its inclusion in multigene cancer panels aimed at investigating the HRR pathway. Since then, various studies reported PVs in 0.25% of BC patients [57], which even included founder variants [58, 59]. As mutation carriers had variable BC features, Zuntini et al. [57] investigated and, together with the Breast Cancer Association Consortium [60], concluded that most of the PVs in *NBN* are proven neutral by segregation analyses, case-control studies and/or expression studies. This resulted in the National Comprehensive Cancer Network (NCCN) guidelines not including *NBN* in their consensus document for BC risk management [61]. The variant is therefore considered LP with regards to Nijemen breakage syndrome, but a variant of unknown clinical significance for BC risk. For this reason, the three offspring of the index patient were only screened for the PV in *BRCA1* (Table 1) without disclosing the potential risk for Nijemen breakage syndrome, an incidental finding. Although carrying a single LP/PV *NBN* variant might not be solely responsible for the disease in *Case 5*, it may act as a co-contributor in the case of a DH as the absence of both the MRN complex (of which NBN forms a critical part) and the *BRCA1* protein will disrupt HRR.

### *ATM* and *BRCA2 *- *Case 6*

The *ATM* protein is a serine-threonine protein kinase that primarily responds to DNA damage once activated by auto-phosphorylation in the presence of a DSB. It converts the inactive dimers into catalytically active monomers [62], which are recruited to the DNA repair site by the MRN complex [63], a critical step for the activation of *ATM* [64]. This, in turn, affects the phosphorylation of *BRCA1*, *CHEK2*, and p53, all involved in a signaling cascade that responds to DSBs.

The *ATM* and ATR (*ATM*- and Rad3-related) kinases are the central regulators of the DNA damage response (DDR) signaling pathway [65]. Although ATM is primarily activated by DSBs, both are activated upon DNA damage and replication stress. These kinases, together with the DNA-PKcs (DNA-dependent protein kinase) kinases, form part of the most upstream DDR kinases, and share a similar domain organization with conserved FAT and FAT carboxy-terminal (FATC) domains flanking their carboxyl termini [66]. These domains are auto-phosphorylated in or near their FAT domains upon damage, which regulates their activation.

ATM(NM_000051.4):c.7978G > T (p.Glu2660Ter) occurred between the conserved FAT domain (spanning aa 1960–2566) and the PI3K/PI4K and FATC domains (aa 2712–2962 and 3024–3056). The premature truncation due to the introduction of a stop codon will deliver a non-functional peptide which will result in protein instability due to markedly reduced kinase activity [67, 68].

The splice-site variant (BRCA2 (NM_000059.4):c.516G > A, p.Lys172 =) will result in the absence of all BRC repeats responsible for the binding of RAD51, together with additional critical domains downstream involved in HRR [69]. In normal circumstances, BRC3/BRC4 binds RAD51 to assist in blocking nucleoprotein filament formation by RAD51. During a DSB, the RAD51-ssDNA nucleoprotein filament engages with duplex DNA to find a sequence homologous to that of the bound ssDNA [70]. Upon location of a homologous segment, the presynaptic filament catalyzes the formation of a DNA joint between the bound DNA molecules, resulting in RAD51-mediated DNA strand invasion and exchange. Their absence is critical as certain tumors with faulty HRR mechanisms may rely on PARP-mediated DNA repair for survival and are sensitive to its inhibition [71].

## Clinical relevance

Most of the tumors observed were TNBC, with *Case 5* diagnosed with bilateral synchronous disease. This type is aggressive and associated with a high risk of mortality due to high relapse rates and poor overall survival. Treatment is challenging and relies upon tumor markers, with treating physicians often not taking the genetic result into consideration. This scenario is slowly changing as more patients in SA now have access to genetic screening through the NHLS.

Available treatment options include immunotherapy, radiotherapy, surgery, and chemotherapy, with chemotherapy being the most common (Table 1). This high-risk disease is generally treated with systemic chemotherapy using anthracyclines, alkylating agents, and taxanes in neoadjuvant or adjuvant settings [72]. According to Han et al. [73], neoadjuvant chemotherapy is the preferred approach to treat stage II or III TNBC. The group also postulates that the addition of pembrolizumab to taxane-platinum-based chemotherapy followed by anthracycline significantly increased pCR rates and improved survival in early-stage TNBC [73].

There are, however, limitations that revolve around the development of resistance to anticancer drugs and off-target toxicity, as evident from *Case 3*. Chemo-resistant TNBC disease is genetically very diverse and evolving, which challenges healthcare practitioners to individualize treatment for incomplete responders and patients relapsing [74]. Advances have been made in the development of Food and Drug Administration-approved drugs that either target programmed cell death or involve antibody–drug conjugates [74]. By implementing personalized medicine aimed at key genetic variants involved in specific molecular pathways, these tumors can be targeted using these inhibitors as single agents and/or in combination with standard chemotherapy regimens. Therefore, professionals across healthcare disciplines should work together to develop effective treatment options for TNBCs. Currently, the mainstay of early-stage TNBC includes systemic chemotherapy and surgery, with or without radiation therapy [73].

Confirming a diagnosis of HBOC is critical to both the patient and related at-risk family members where clinical management is concerned. According to the NCCN guidelines, screening options for *BRCA1/2* mutation carriers should include bi-annual clinical breast examinations with magnetic resonance imaging (MRI) or mammogram annually from age 25, as well as prophylactic risk-reducing bilateral mastectomy with or without reconstruction. For carriers with LP/PVs in the moderate penetrance genes such as *ATM*, annual screening with mammography or MRI is recommended from age 40 with a prophylactic mastectomy only if the family history warrants it. In cases where there is also a strong OVC association, the guidelines recommend a prophylactic risk-reducing BSO from the age of 40 years (Table 2). There is scarce literature documenting the clinical management of DHs, as these patients are managed for the combined cancer burden across both genes. When both genes confer a risk for the same cancer type, the patient is managed according to the guidelines associated with the gene that conveys the highest absolute risk. At-risk related family members should be offered predictive genetic testing for both LP/PVs involved if considered clinically actionable. In the instance of *Cases 4&5*, predictive genetic testing for *NBN* and *FANCD2* is currently not clinically indicated according to the guidelines due to their unknown associated risk and a lack of evidence regarding clinical management for BC and OVC (Table 2).

**Table 2:** Cancer risks and recommended management options associated with the genes identified in six DH mutation carriers according to the latest NCCN guidelines, Version 3.2024 [63] Can we move columns 4 to 6 more to the right to ensure that Columns 2 and 3 are less cramped?

Gene	Absolute risk of female BC (%)	Other associated cancers	Age of recommended BC screening (MRI/mammogram)	Is risk-reducing mastectomy recommended?*	Is risk-reducing salpingo-oophorectomy recommended?*
*BRCA1*	> 60	Ovarian, pancreatic, prostate, male BC	25 years	Yes	Yes
*BRCA2*	> 60	Ovarian, pancreatic, prostate, male BC, melanoma	25 years	Yes	Yes
*TP53*	> 60	Soft tissue sarcoma, osteosarcoma, CNS tumor, ACC, melanoma, colorectal, prostate, gastric, pancreatic ca	20 years	Yes	n/a
*ATM*	20–30	Pancreatic, prostate, ovarian ca	30 years	No	No
*BARD1*	17–30	Unknown or insufficient evidence	40 years	No	n/a
*FANCD2*	Unknown or insufficient evidence	Unknown or insufficient evidence	n/a	n/a	n/a
*NBN*	Unknown or insufficient evidence	Unknown or insufficient evidence	n/a	n/a	n/a

BC - breast cancer, CNS - central nervous system, ACC - adrenocortical carcinoma, ca - cancer, n/a - not applicable

*Based on molecular diagnosis alone. Surgery can be considered for all cases with a significant family history of the disease

Genetic counseling is an important resource for affected individuals and their families, as counselors specialize in helping individuals understand their hereditary risk and facilitate informed, often emotional, decision-making during testing. Post-test counseling of these women and their related family members is crucial to ensure that patients and healthcare providers understand the risks and management associated with genetic conditions. For DHs, counseling on recurrence risks will be more complex as more than one gene might be involved, with some individuals inheriting one or both or neither of these variants.

The mutational status of the DHs carrying LP/PVs in genes involved in the HRR machinery (such as *BRCA1/2*, *TP53*, and *ATM*) has implications for the use of adjuvant radiotherapy (ART). As DNA damage will occur due to ART, it will severely impact cell cycle recruitment, proliferation, and apoptosis, resulting in impaired DSBs, consequently increasing the risk of second malignant neoplasms occurring [75–77]. Unfortunately, the genetic results of *Cases 1&4* were not yet available at the time of treatment planning. Therefore, knowledge of their germline *TP53* variants could not be considered during treatment planning.

The management of patients with especially germline *TP53* (*Cases 1&4*) variants is complex as clinical decision-making needs to weigh the risks of normal tissue toxicity and radiosensitivity against the tumor’s response (radioresistance and radio curability) [78]. Although ART should be avoided as far as possible in *TP53*-associated disease, according to the guidelines of the American Society for Radiation Oncology (ASTRO) and the American Society of Clinical Oncology (ASCO) [79], the overall prognosis might be poor. Since *Case 4* presented with locally advanced BC (stage IIIb), a strong indication for post-mastectomy ATR [79], knowledge about the *TP53* variant would not have affected the treatment decision of ATR for this patient at risk of locoregional disease relapse. Despite the contraindication for ART based on the detection of a pathogenic germline *TP53* variant [76–78], the tumor stage and histopathology results can override the risk associated with a germline *TP53* variant, as evident from achieving pCR post-therapy (Table 1).

## Conclusion

This study detected a low frequency (0.375%) of bi-allelic BC patients in the SA population, driven by *BRCA1/2* founder variants. Additionally, family-specific variants were detected in *ATM, BARD1, FANCD2, NBN*, and *TP53*. Although the scientific interpretation and clinical relevance were based on the clinical and tumor characteristics of the DHs, the detection of an incidental NBN variant caused a dilemma for report writing and genetic counseling regarding family follow-ups. The study highlighted the potential consequences of treatment initiation that preceded genetic test results due to sample batching to reduce laboratory costs. This resulted in patients potentially receiving risk-enhancing treatments such as radiotherapy associated with induced risk in *TP53* carriers. Despite the small sample size, valuable insights were gained, which highlighted the importance of tumor pathology in bringing genomics into the clinical domain [80]. There is a need for harmonious cooperation between geneticists and clinicians to address multiple challenges associated with the merging of traditional boundaries between diagnosis and treatment in cancer management.

## Data Availability

The genetic data referred to in this publication is available on ClinVar (submission number SUB11378633 (SCV002504693–SCV002505346)).
